# Exploratory study of autophagy inducer sirolimus for childhood cerebral adrenoleukodystrophy

**DOI:** 10.3389/fped.2023.1187078

**Published:** 2023-06-06

**Authors:** Xiao-Mei Luo, Li-Ying Liu, Qiu-Hong Wang, Yang-Yang Wang, Jing Wang, Xiao-Yan Yang, Shi-Jun Li, Li-Ping Zou

**Affiliations:** ^1^ Senior Department of Pediatrics, Medical School of Chinese PLA, Chinese PLA General Hospital, Beijing, China; ^2^ Department of Pediatrics, Guangzhou Women and Children's Medical Center, Guangdong Provincial Clinical Research Center for Child Health, Guangzhou, China; ^3^ Department of Pediatrics, Xuanwu Hospital Capital Medical University, Beijing, China; ^4^ Department of Neurology, Beijing Jingdu Children's Hospital, Beijing, China; ^5^ Department of Radiology, First Medical Center, Chinese PLA General Hospital, Beijing, China

**Keywords:** childhood cerebral adrenoleukodystrophy, autophagy, sirolimus, white matter hyperintensities, demyelination

## Abstract

**Objectives:**

X-linked adrenoleukodystrophy (ALD) is a peroxisomal disease caused by mutations in the *ABCD1* gene. Childhood cerebral ALD (CCALD) is characterized by inflammatory demyelination, rapidly progressing, often fatal. Hematopoietic stem cell transplant only delays disease progression in patients with early-stage cerebral ALD. Based on emergency humanitarianism, this study aims to investigate the safety and efficacy of sirolimus in the treatment of patients with CCALD.

**Methods:**

This was a prospective, single-center, one-arm clinical trial. We enrolled patients with CCALD, and all enrolled patients received sirolimus treatment for three months. Adverse events were monitored and recorded to evaluate the safety. The efficacy was evaluated using the neurologic function scale (NFS), Loes score, and white matter hyperintensities.

**Results:**

A total of 12 patients were included and all presented with CCALD. Four patients dropped out and a total of eight patients in the advanced stage completed a 3-month follow-up. There were no serious adverse events, and the common adverse events were hypertonia and oral ulcers. After sirolimus treatment, three of the four patients with an initial NFS > 10 showed improvements in their clinical symptoms. Loes scores decreased by 0.5–1 point in two of eight patients and remained unchanged in one patient. Analysis of white matter hyperintensities revealed a significant decrease in signal intensity (*n* = 7, *p* = 0.0156).

**Conclusions:**

Our study suggested that autophagy inducer sirolimus is safe for CCALD. Sirolimus did not improve clinical symptoms of patients with advanced CCALD significantly. Further study with larger sample size and longer follow-up is needed to confirm the drug efficacy.

**Clinical Trial registration:** https://www.chictr.org.cn/historyversionpuben.aspx, identifier ChiCTR1900021288.

## Introduction

Autophagy is a tightly regulated intracellular lysosomal pathway involved in the breakdown and removal of damaged cellular components and debris. Autophagic deficiency may be one of the important pathological features underlying many neurodegenerative diseases in patients (1). In recent years, findings from a large number of studies have demonstrated a strong link between disruptions in autophagic pathways and various neurodegenerative diseases such as Parkinson's disease, Alzheimer's disease, Huntington's disease, and even some leukoencephalopathies (2–6).

X-linked adrenoleukodystrophy (ALD) is a rare neurodegenerative disease that results in the rapid inflammatory demyelination and axonal degeneration, and pathogenic mutations in ABCD1 (ALD) gene located on chromosome Xq28 has been identified as the etiology (7, 8). Despite this, the exact pathogenic mechanism of ALD is still not known. Previous research suggested that a variety of functional disorders caused by the excess of very long chain fatty acids (VLCFA), such as defective mitochondrial biosynthesis targeting the SIRT1/PGC-1α axis, the ubiquitin-proteasome system malfunction, peroxisomes malfunction, and the aberrant overactivation of the mTOR pathway that inhibits the process of autophagy, may be the primary culprits (9). Childhood cerebral adrenoleukodystrophy (CCALD) is the most serious type, and if not timely treated, cerebral inflammatory demyelination can lead to loss of neurological and cognitive function in early childhood (10, 11).

Hematopoietic stem cell transplantation (HSCT) and hematopoietic stem cell gene therapy are effective only in the early stage of CCALD. For patients with advanced-stage cerebral ALD, no therapeutic interventions are available. Launay et al. reported the key role of autophagy in the pathogenesis of ALD. They found that elevated mammalian target of rapamycin (mTOR) signaling, which is a key regulator of cellular metabolism, growth and survival, led to impaired autophagy. Moreover, they have shown that the mTOR pathway inhibitor and autophagy inducer, temsirolimus, a sirolimus derivative, can restore autophagic flow and inhibits axonal degeneration and associated motor function impairments in the mouse model, playing a protective role by preventing the induction of neuronal apoptosis (12). Other studies have also confirmed the role of sirolimus in anti-oxidative stress and neuroprotection by inducing autophagy (13, 14). By regulating autophagy to rescue neuronal apoptosis may be a new targeted therapy for neurodegenerative diseases. Autophagy inducer sirolimus has been reported to be safe for children. In the study of sirolimus in the treatment of tuberous sclerosis in children, the main adverse events associated with sirolimus were oral ulcers, upper respiratory tract infections, fever, and no serious adverse events (SAE) occurred, which demonstrated the safety of sirolimus (15, 16).

Although the efficacy of sirolimus has been confirmed in the mouse model presenting as adrenomyeloneuropathy, it also provides a basis for the study of cerebral ALD because they are all based on the mechanism of abnormal VLCFA accumulation. Based on emergency humanitarianism, we conducted the prospective, one-arm clinical study in patients with advanced CCALD to evaluate the safety of sirolimus and whether it would be effective in reducing the signs and symptoms.

## Material and methods

### Study design and participants

This was a prospective, single-center, one-arm clinical trial based on emergency humanitarianism. This study was approved by the institutional ethics committee and registered in the Chinese Clinical Trial Registry (Registration Number: ChiCTR1900021288). We enrolled patients with CCALD who were treated at Beijing Jingdu Children's Hospital from February 2019 to October 2019. Each patient's diagnosis was confirmed by biochemical, brain MRI, and genetic testing. Criteria for inclusion were as follows: (1) Males between 6 and 18 years of age (2) Genetic test confirmed ABCD1 gene variation (3) Elevated VLCFA levels (4) Central nervous system disease established by radiological examination of anatomical brain MRI findings (5) A score on neurologic function scale (NFS) > 1 points. We acquired written informed consent from the parents/guardians of all enrolled patients.

The early stage of the CCALD is defined as NFS ≤ 1, Loes ≥ 0.5–≤9, and the advanced stage is defined as NFS > 1, Loes > 9.

For patients who were taking other medications orally, such as hydrocortisone, anti-seizure medications, etc., we did not adjust the dosage of all drugs taken at the time of enrollment, and based on the principle of maximizing the benefits of patients, we did not ask the patients to withdraw the medication they were taking.

### Study procedures

All enrolled patients received sirolimus (Hangzhou Zhongmei Huadong Pharmaceutical Co. Ltd., 50 mg/50 ml) treatment with an initial dose of 1 mg/m^2^/day. Blood concentration levels were monitored during the medication and the target trough blood concentration was 5–10 ng/ml (16, 17). The drug dose would be increased to reach the target concentration. Sirolimus treatment lasted for a total of three months. Patients may request a reduction or withdrawal from the study if treatment-related SAE occurs.

Safety was assessed by the reports of adverse events, physical and neurological examinations. The enrolled patients were taken blood samples to monitor blood routine and blood biochemistry before receiving sirolimus, and were regularly reviewed during treatment. SAE and deaths, regardless of whether the events were considered to be related to sirolimus were collected by the investigator.

Efficacy was evaluated using the primary outcome measures that included the score on NFS, Loes score, and white matter hyperintensities (WMH).

The NFS is evaluated according to the ALD neurologic severity scale ranging from 0 to 25 and is based on the appearance and severity of neurological symptoms (Supplementary Table S1). The higher the score, the more severe the neurological symptoms (11). The NFS evaluation was performed by clinicians unrelated to the study to eliminate subjective bias of the investigator. At each evaluation of the NFS, more than two clinicians were involved, and the same clinicians were used to make assessments at the beginning and end of the study.

Brain MRI were acquired using a Siemens 1.5 T scanner for conventional 12 channels of head coil, and collect patient image information on T1WI, T2WI and Flair sequence. The Loes score is based on an evaluator's ratings of the affected location and severity of a lesion, and whether there is focal and/or general brain atrophy (Supplementary Table S2). A normal score is equivalent to 0 for each area, 0.5 for unilateral involvement, with 1 for bilateral lesions or atrophy. Loes score ranges from 0 to 34, with higher scores indicating an increased extent of lesions on brain MRI (18). The brain MRI assessment was done by two independent radiologists. For the Loes score with inconsistent results, a third radiologist will be invited to evaluate and finally come to an agreed result. The same radiologists were used to make assessments at the beginning and end of the study. In addition, all radiologists who participated in the evaluation were not aware of the prior Loes score of the enrolled patients or whether they were involved in the intervention study.

We have used the Loes rating system to evaluate the severity of MRI lesions. However, it is a subjective measure and cannot reliably detect changes in the volume of WMH in the brain. To compare the difference of WMH before and after treatment, we linearly aligned the MRI enhancement with Gadolinium (Gd) pre- and post-treatment for each participant using FLIRT (19, 20) from FSL v6.0 (https://fsl.fmrib.ox.ac.uk/fsl/fslwiki/FSL). For each patient, the WMH region of interest (WMH-ROI) of white-matter was extracted through a manual thresholding method. Specifically, an experienced radiological technician manually set individual-specific threshold for the WMH-ROI by visual inspection. The thresholds were adapted for each patient to include possibly all WMH voxels and exclude voxels of other high-intensity types, such as blood vessels. However, parts of vascular voxels were inevitably included in the WMH-ROIs due to partial overlapping of signal intensities. Although each WMH-ROI inevitably contained less or more vascular signals, the impact on the following analysis could be limited taking into account of the relatively small proportions of vascular voxels compared to WMH-ROIs.

### Statistical analysis

All statistical analyses were performed by using statistical software SPSS v. 22.0. Continuous variables were described by median, maximum and minimum values. Descriptive statistics were used to describe the Loes score and NFS score. In order to quantify the changes of WMH, we firstly combined the WMH-ROIs extracted from the images before and after treatment of each patient. For each patient, we then calculated the mean and standard deviation of signals within the conjunct ROI for images pre-and post-treatment, separately. Next, a non-parametric paired test (Wilcoxon signed rank test) was used to investigate whether the WMH in conjunct ROIs was significantly different before and after treatment.

## Results

### Baseline data

**Figure 1:**
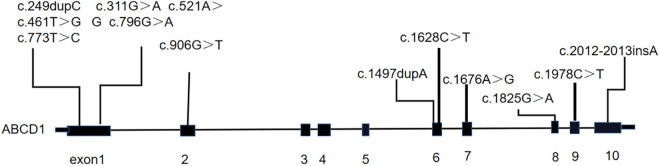
The location of mutation site for each patient on *ABCD1* gene.

A total of 12 male patients with CCALD were enrolled (Table 1). The median age of disease onset and enrollment was 82 months (61–117 months) and 88.5months (74–155 months), respectively. All patients were found to carry ABCD1 gene mutations via genetic testing, of which nine were missense mutations and three were frameshift mutations. The locations of the gene mutation sites are shown in Figure 1. The mutation sites were maternal in 11 patients, and only one was *de novo* (Table 1). Two of the 12 patients had a known family history of CCALD. At baseline, each patient underwent neurological functional assessment using the NFS and Loes score. The NFS scores ranged from 3 to 22 points with the median of 16.5, and the Loes scores ranged from 14.5 to 27 points with the median of 19. All patients were in the advanced stage.

**Table 1:** Baseline characteristics of ALD patients and changes after sirolimus treatment.

Number	Gender (F/M)	Age (m)	Oneset age (m)	Diagnosed age (m)	Family history (Y/N)	*ABCD1* mutation site	Mutation type	Mutation source	NFS score		Loes score		VLCFA					
													Pre-treatment			After sirolimus treatment		
									Pre-treatment	After sirolimus treatment	Pre-treatment	After sirolimus treatment	C26:0 (μmol/L)	C26:C22	C24:C22	C26:0 (μmol/L)	C26:C22	C24:C22
01	M	123	117	117	Y	c.773T>C	Missense	Maternal	4	11	14.5	13.5	2.85	0.057	1.51	2.61	0.044	1.27
02	M	74	61	67	N	c.2012-2013insA	Frameshift	Maternal	19	16	17.5	19.5	3.96	0.083	1.75	3.84	0.085	1.81
03	M	82	70	76	N	c.311G>A	Missense	Maternal	21	18	19	19.5	2.75	0.056	1.35	3.21	0.075	1.62
04	M	89	83	88	N	c.1628C>T	Missense	Maternal	3	11	15	15	1.58	0.046	1.27	1.69	0.041	1.07
05	M	80	70	70	N	c.1978C>T	Missense	Maternal	15	16	19.5	19	4.47	0.077	1.62	4.51	0.095	1.5
06	M	88	68	69	N	c.1817C>T	Missense	Denovo	22	NA	20.5	NA	3.57	0.076	1.48	NA	NA	NA
07	M	100	90	95	N	c.521A>G	Missense	Maternal	6	12	19.5	21	3.76	0.075	1.33	4.44	0.073	1.57
08	M	74	72	73	N	c.1676A>G	Missense	Maternal	3	10	14.5	18	5.17	0.082	1.74	4.87	0.088	1.63
09	M	98	88	88	Y	c.796G>A	Missense	Maternal	21	20	22.5	23.5	2.95	0.074	1.52	4.39	0.106	1.76
10	M	125	123	124	Y	c.796G>A	Missense	Maternal	0	NA	2.5	NA	2.12	0.051	1.19	NA	NA	NA
11	M	73	81	82	N	c.1825G>A	Missense	Maternal	9	NA	19	NA	2.73	0.101	1.43	NA	NA	NA
12	M	132	102	116	N	c.249dupC	Missense	Maternal	20	NA	21	NA	3.76	0.056	1.53	NA	NA	NA
13	M	155	88	98	N	c.1497dupA	Frameshift	Maternal	18	NA	27	NA	1.33	0.044	1.08	NA	NA	NA

F, Female; M, Male; m, month; Y, Yes; N, No; NFS, Neurological Function Score; VLCFA, Very Long Chain Fatty Acids; NA: Data unavailable.

### Safety

We continuously monitored all adverse events that occurred during the treatment, whether they were related to sirolimus or not (Table 2). Five of 12 patients experienced aggravation of hypertonia after oral sirolimus, of which three patients withdrew. Three patients had oral ulcers during medication. Two patients showed signs of a fever during the course of treatment, and soon returned to normal after active symptomatic anti-infection treatment. Two patients developed status epilepticus during the treatment and seizures were controlled soon after sedation and anticonvulsant therapy. The other patients had no significant adverse events during the treatment.

**Table 2:** Adverse events during medication.

Adverse events	*N* (%)
Aggravation of hypertonia	5 (38.5%)
Oral ulcers	3 (23.1%)
Fever	2 (15.4%)
Status epilepticus	1 (7.7%)

The occurrence of oral ulcers was related to sirolimus, and through drinking more water and gargling to reduce the drug residues in the mouth, the occurrence of oral ulcers reduced. The occurrence of status epilepticus was considered as one of the clinical symptoms in the progression of ALD. The aggravation of hypertonia has the highest incidence of all adverse events, and when oral ulcers became severe, the hypertonia aggravated because of the pain, so it may be related to the use of sirolimus. No SAE or deaths were observed.

### Efficacy evaluation

Of all the 12 enrolled patients, eight patients in the advanced stage received sirolimus treatment and completed the 3-month follow-up. Four patients dropped out, including one (patient 13) who did not complete follow-up, three (patient 6, 11, 12) who discontinued the treatment due to severe hypertonia after oral sirolimus treatment.

**Figure 2:**
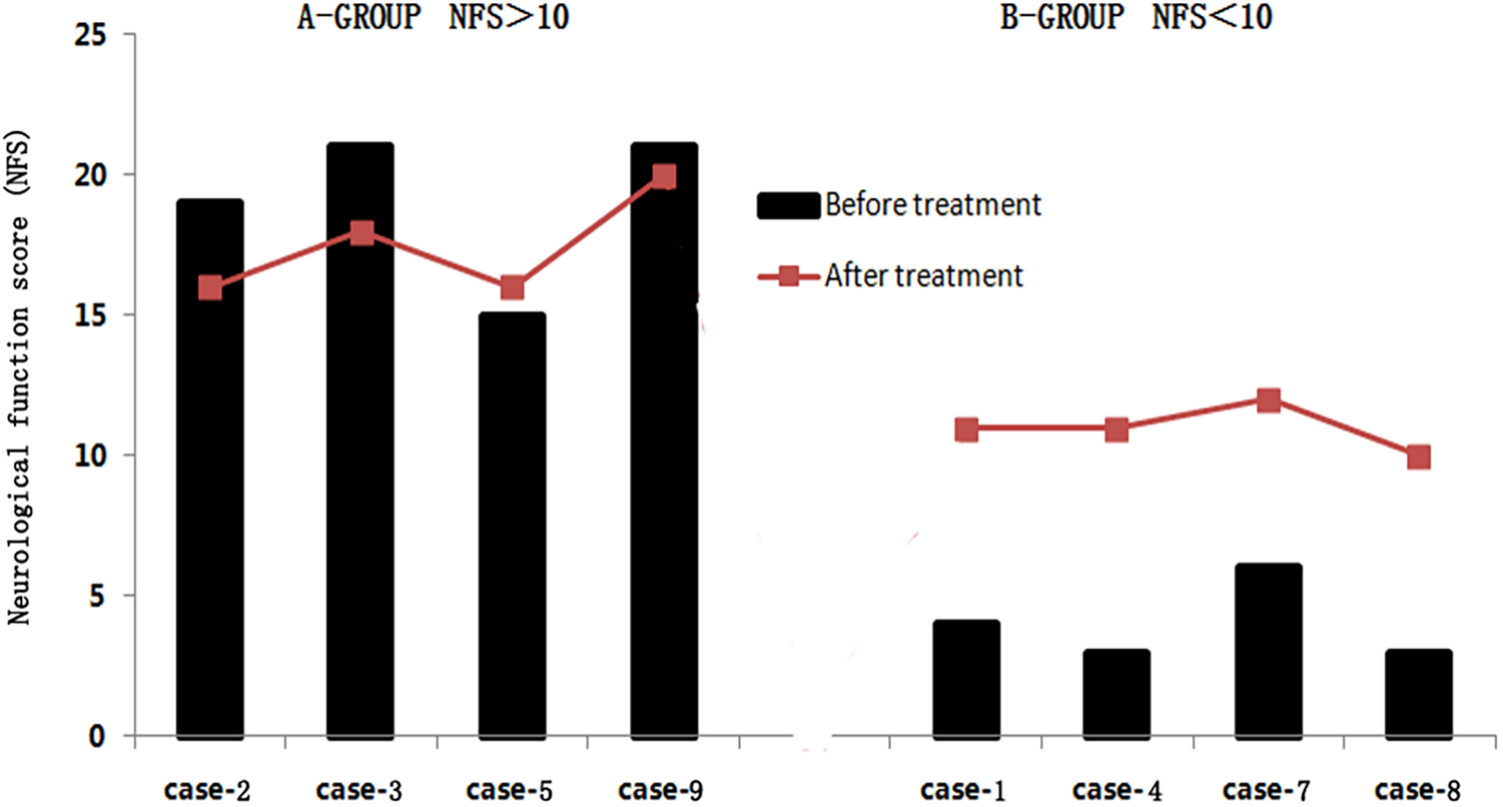
Changes of neurological function score (NFS) by sirolimus-treated patients. A-Group: NFS > 10. B-Group: NFS < 10.

Among the eight patients, there were four patients with the NFS score > 10 and another four patients < 10. In the research, we found that after sirolimus treatment, three of the four patients (NFS score > 10) achieved improvement in the clinical symptoms, with NFS scores decreasing by 1–3 points and one patient increased 1 point, while the other four patients (NFS score < 10) were still progressing, with NFS scores increasing by 1–8 points (Figure 2).

**Figure 3:**
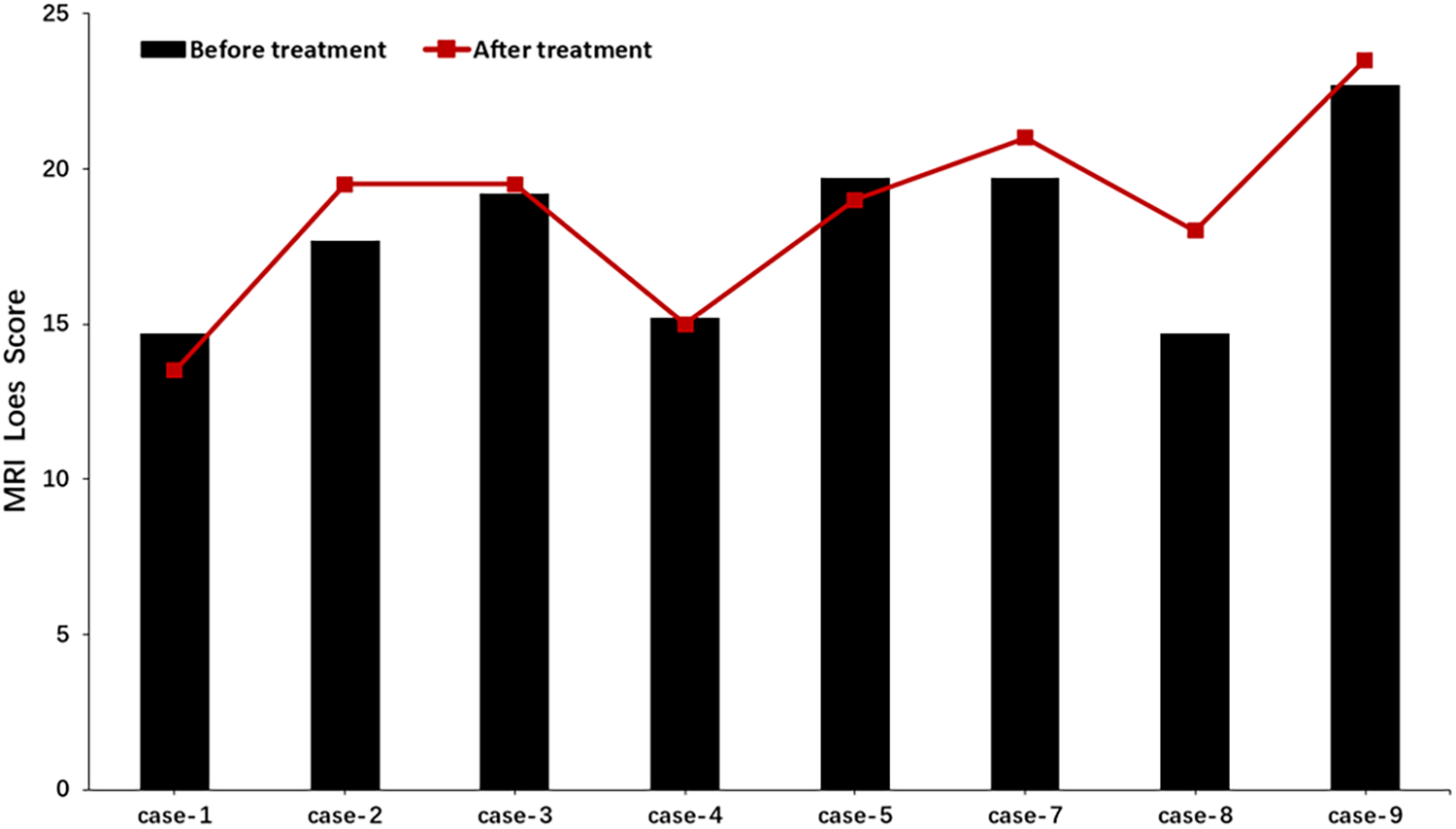
Changes of MRI Loes score before and after treatment by sirolimus-treated patients.

**Figure 4:**
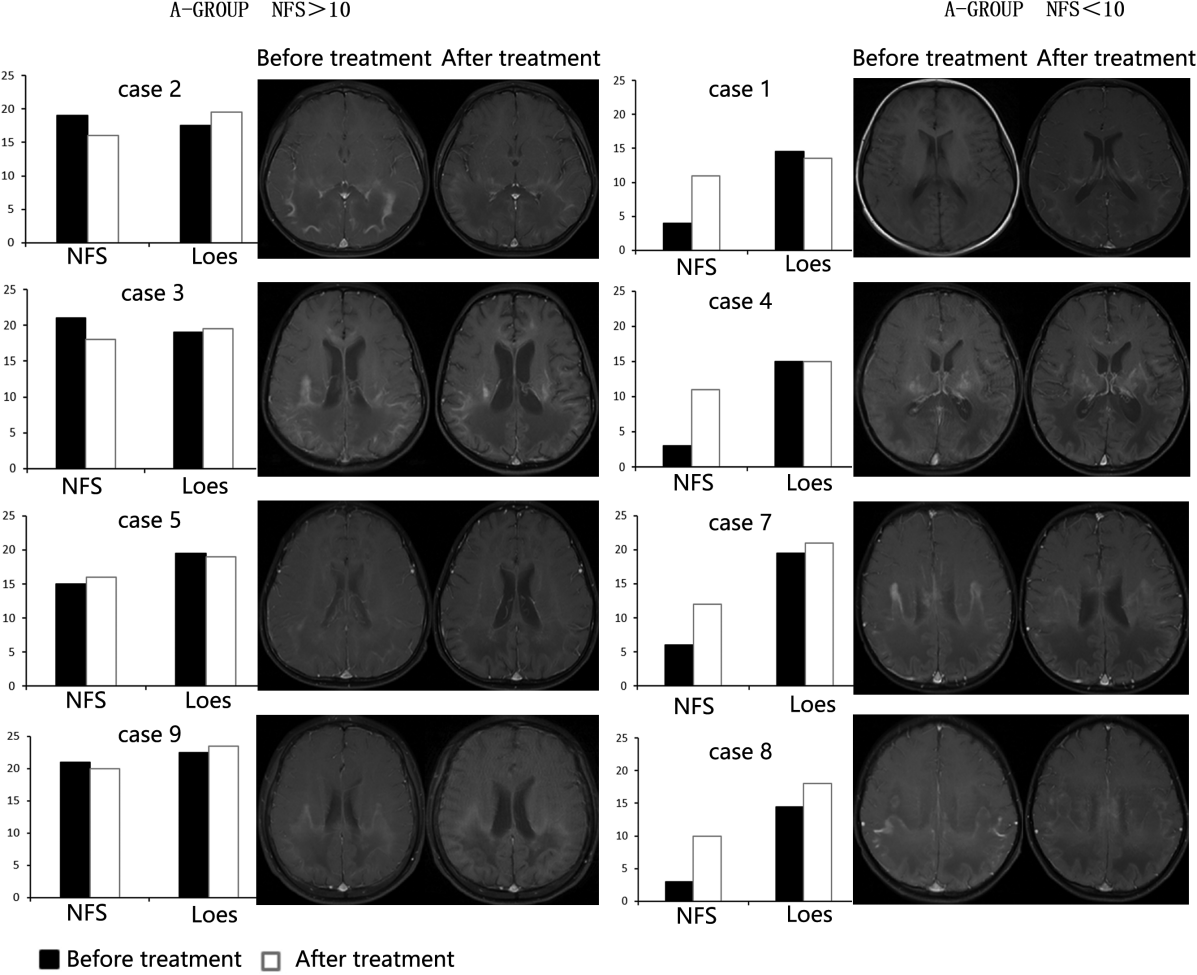
The MRI images after processed (left: before treatment, right: after treatment; case 5 could not be compared due to inconsistent scanning parameters before and after treatment and was excluded.).

**Figure 5:**
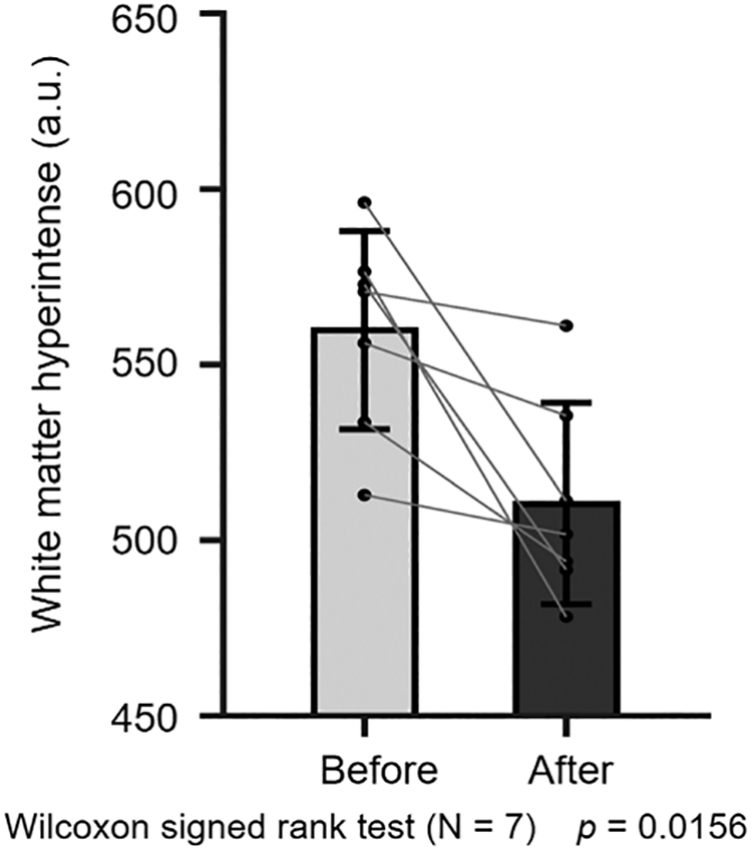
Statistical comparison of white-matter hyperintensity before and after treatment (*n* = 7, *p* = 0.0156).

After three months of treatment, as measured with the Loes score, two of eight patients decreased by 0.5–1 point, and one patient remained unchanged, and the other five increased by 0.5–3.5 points (Figure 3). Considering that the Loes score cannot accurately reflect the changes in the volume of WMH, we processed the MRI images to better evaluate the change of WMH in the brain. Because patient 1 could not obtain the consistent scanning parameters before and after treatment, seven patients participated in the comparison of WMH in the brain. The results of processed image are shown in Figure 4. The statistical results of WMH are shown in Table 3. The difference in the volume of WMH before and after treatment in seven patients was statistically significant (*p* = 0.0156) (Figure 5).

**Table 3:** Statistical results of white-matter hyperintensity before and after treatment.

Subjects	White-matter hyperintensity before treatment (mean ± std)	White-matter hyperintensity after treatment (mean ± std)
Patient 2	533.51 ± 76.70	493.96 ± 73.35
Patient 3	596.10 ± 81.11	511.20 ± 90.32
Patient 4	556.06 ± 67.61	535.41 ± 86.49
Patient 5	512.87 ± 70.61	501.65 ± 83.65
Patient 7	570.71 ± 76.60	561.05 ± 91.58
Patient 8	572.78 ± 84.75	491.57 ± 82.74
Patient 9	576.44 ± 60.16	478.15 ± 59.14

## Discussion

In this study, sirolimus was used to treat patients in the advanced stage of CCALD based on emergency humanitarianism. Our results showed that the autophagy inducer sirolimus could be safe for patients with CCALD, and the efficacy of sirolimus in improving the clinical symptoms of advanced ALD requires further studies with larger sample size and long-term follow-up.

CCALD can lead to death in young childhood. HSCT and hematopoietic stem cell gene therapy are only effective at an early stage, and there are no other effective treatments for CCALD. Moreover, there are risks of transplant failure and complication of the conditioning regimen (21, 22). In addition, the disease of CCALD progresses rapidly, and patients often have a rapid spread of demyelination in the brain, with death in a few years (23). Given the rapid progression of the disease and the absence of a treatment option to stop progression, and based on the possible efficacy of sirolimus in X-ALD proposed by Launay et al. in 2014, we tried this treatment regimen clinically. All enrolled patients were in the advanced stage and did not meet the criteria for HSCT and hematopoietic stem cell gene therapy, and these patients had no other treatment options.

No serious life-threatening adverse events were found, and the biochemical indicators remained stable, indicating that sirolimus is relatively safe for treating CCALD. Fever was considered to be a common side-effect of sirolimus. Three patients did develop the aggravation of hypertonia after sirolimus treatment, and the mechanism is not entirely clear. We cannot definitively assume that it was a direct sirolimus-related adverse event, because these patients also had oral ulcers caused by sirolimus. Pain associated with oral ulcers may also cause exacerbation of hypertonia. No other SAE occurred, which to some extent, indicated the safety of sirolimus in CCALD patients.

Sirolimus, as an autophagy inducer, can inhibit the mTOR pathway, reduce axonal degeneration, and arrest disease progression in Abcd1−/Abcd2−/− mice (12). Among patients with NFS score < 10, there was progress in scoring, which was thought to be related to the shorter follow-up period. Among patients with NFS score >10, we found an improvement of 1–3 points. However, the follow-up time was shorter, so we cannot determine the impact on quality of life. At present, the reported effective treatments of CCALD are HSCT and hematopoietic stem-cell gene therapy. However, they are only effective in patients in the early stage of CCALD (21, 22). Based on these studies, we considered whether sirolimus, like HSCT and hematopoietic stem cell gene therapy, is only effective against CCALD in the early stage. However, the CCALD patients enrolled in our study were all in the advanced stage. Therefore, whether sirolimus is effective for ALD patients in the early stage of the disease requires further long-term follow-up studies with large sample size.

Brain MRI is an important method of diagnosing ALD. Neuroimaging studies have shown that the MRI pattern of demyelination in ALD is related to disease progression and outcome (24), and the progress of Loes score is also closely related to survival rate (24, 25). Our results showed that the more severe the clinical symptoms, the higher the Loes score; that is, the severity of brain MRI is related to the severity of clinical symptoms. This is consistent with previous research. In addition, Loes scores declined or stabilized in three of the eight patients, which also provides some hope for patients in the advanced stage without effective regimens. However, the sample size of this study is small, which needs to be confirmed by further studies. The traditional Loes score is evaluated according to the affected area, but it cannot accurately reflect the changes in the volume of WMH. So, we quantitatively analyzed the WMH, and finally found that the volume of WMH decreased after sirolimus treatment, and the difference was statistically significant. This suggests that the relationship between sirolimus and demyelination in the brain deserves further attention. In addition, it has been reported that longitudinal changes in WMH volume were progressive in most patients (26, 27). Some mild patients may have multiple possibilities for the changes in WMH volume: progression, regression, or stability, and most of the regression is related to the intervention (28). In this study, the patients were in advanced stage of disease, so it is unlikely that a reduction in WMH would have occurred without intervention. After sirolimus treatment, WMH volume decreased and the difference was statistically significant, suggesting the role of sirolimus to some extent. However, the analysis of WMH has certain limitations. There seem progressive atrophic changes in the brain including the white matter, as a result, comparison areas of WMH may represent atrophic changes. Therefore, the effect of brain atrophy on WMH volume change cannot be ruled out.

For the pathogenic mechanism, it has been reported that inflammatory demyelination of cerebral ALD seems to be related to autoimmunity, such as increased serum antibody responses against myelin oligodendrocyte glycoprotein (29, 30). The mTOR inhibitor sirolimus has shown remarkable clinical efficacy in autoimmune diseases such as systemic lupus erythematosus and multiple sclerosis (31, 32). Therefore, it would be of interest to monitor the immunological status, such as routine detection of antinuclear and myelin basic protein and myelin oligodendrocyte auto-antibodies to demonstrate efficacy of sirolimus in patients with ALD. However, the limitation of this study is that patients' immune status was not monitored during treatment. We expect to complete the detection of autoantibody status in future studies.

Glucocorticoids have been reported to inhibit inflammation (33). In this study, hydrocortisone treatment was performed in 2 of the 8 patients who completed the study, however, it is worth noting that, the 2 patients had been taking hydrocortisone (0.22–0.49 mg/kg/day) for a long time before enrollment. In addition, the original dose of the drug did not change after enrollment, which reduced the influence of hydrocortisone to some extent.

Although it is strictly designed, it still has some limitations in this study. First, there was no control group in this study. Since all of the patients enrolled in this study are CCALD in the advanced stage with rapid disease progression, and death can occur within several years. Moreover, their parents were willing to take various treatments for their children instead of waiting, so this study based on emergency humanitarianism treated CCALD patients in the advanced stage of the disease with sirolimus. In addition, for patients who received HSCT previously, they were all at an early stage, so no suitable historical control has been found. Based on the above reasons, this study was set as a self-controlled study of sirolimus add-on therapy, and all patients maintained their original oral drug dose after enrollment. Second, it is based on the principle of maximizing the benefits of patients, so during the treatment, all patients did not change their original treatment drugs, which led to bias of research results, and the interference of drug interactions could not be ruled out. The last, since sirolimus was used for the first time in ALD patients, we designed this research as a pilot study in consideration of the safety, so the sample size is small and the follow-up time of three months is relatively short, and it was not clear whether sirolimus could benefit ALD patients in the long term. Therefore, it is necessary to further study the efficacy, optimal dose, treatment duration, and indications of sirolimus in larger randomized controlled trials. Despite this, our study suggests that sirolimus could be a safe choice for CCALD patients.

## Conclusion

The autophagy inducer sirolimus may be safe to treat CCALD patients. However, larger sample sizes and longer follow-up studies are needed to clarify the efficacy of sirolimus in patients with advanced CCALD.

## Data Availability

The raw data supporting the conclusions of this article will be made available by the authors, without undue reservation.
