# Supplementary material for: Exploratory study of autophagy inducer sirolimus for childhood cerebral adrenoleukodystrophy
Source: Front Pediatr. 2023 Jun 6;11:1187078. doi: 10.3389/fped.2023.1187078 (PMC10289280; doi:10.3389/fped.2023.1187078)
Supplement: Supplementary file 1 [file Table1.docx]

| Table S1. Neurologic function scale score | |
| --- | --- |
| symptoms | score |
| auditory processing disorder | 1 |
| aphasia/apraxia | 1 |
| loss of communication | 3 |
| visual impairment | 1 |
| blindness | 2 |
| dysphagia | 2 |
| tube feeding | 2 |
| dyspraxia/hyperreflexes | 1 |
| difficulty walking/tetanic state/spasmodic gait (without assistance) | 1 |
| spasmodic gait (require assistance) | 2 |
| loss of mobility | 2 |
| loss of movement | 3 |
| paroxysmal Incontinence | 1 |
| total Incontinence | 2 |
| non-febrile convulsion | 1 |
| total | 25 |

|  | | |
| --- | --- | --- |
| Table S2. Loes Score | | |
| Major categories | Subdivisions | Score (maximum) |
| Parietooccipital white matter |  | 4 |
| Anterior temporal white matter | periventricular, central, subcortical | 4 |
| Frontal white matter |  | 4 |
| Corpus callosum | splenium, body, genu | 5 |
| Visual pathway | optic radiations, meyer's loop, lateral geniculate body | 4 |
| Auditory pathways | medial geniculate body, brachium to the inferior colliculus, lateral lemniscus, pons | 4 |
| Frontopontine-corticospinal projection fibers | internal capsule, brain stem | 2 |
| Cerebellum | white matter, atrophy | 2 |
| Basal ganglia | — | 1 |
| Global atrophy, | mild, moderate, severe | 4 |
| Total | — | 34 |
